# Two-step generation of monodisperse agarose-solidified double emulsions (w/w/o) excluding an inner oil barrier

**DOI:** 10.1016/j.mex.2021.101565

**Published:** 2021-11-02

**Authors:** Stephan Brinkmann, Markus Oberpaul, Jens Glaeser, Till F. Schäberle

**Affiliations:** a Fraunhofer Institute for Molecular Biology and Applied Ecology (IME), Branch for Bioresources, 35392 Giessen, Germany; b Evotec International GmbH, Göttingen 37079, Germany; c Institute for Insect Biotechnology, Justus-Liebig-University of Giessen, Giessen 35392, Germany

**Keywords:** Droplet-based microfluidics, Water-in-water-in-oil emulsion, Microreactor, Microencapsulation, Microdroplet, High-throughput assay

## Abstract

Miniaturization of biomedical and chemical research areas is performed using microfluidic techniques. Droplet-based microfluidic applications are of high interest for various applications, e.g., high-throughput screening assays. Many of them are based on simple water-in-oil (w/o) or oil-in-water (o/w) emulsions that are easily to produce. More complex assays based on separate compartments require the use of multiple emulsions, such as water-in-oil-in-water (w/o/w) or oil-in-water-in-oil (o/w/o) emulsions. In this study an easy, fast to establish method to generate agarose-solidified (w/w/o) double emulsions with ∼55 µm in diameter, in which both agarose-phases are not separated by a surfactant stabilized oil is described. An off-chip emulsion-breaking and washing step of the inner agarose droplets based on density gradient centrifugation was designed, offering new possibilities for high-throughput assays on picoliter scale. In brief, this paper reports:

• the protocol to generate agarose-solidified (w/w/o) double emulsions non-seperated by surfactant stabilized oil;
• an off-chip washing protocol of agarose-solidified emulsions based on density gradient centrifugation.

## Graphical abstract

**Figure:**
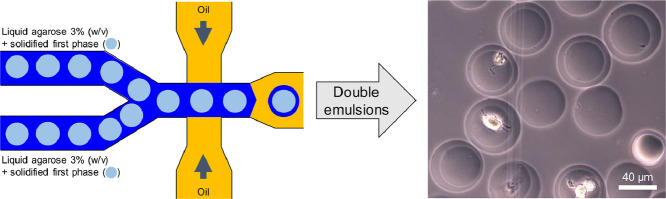

Specifications table

**Table:** 

Subject Area:	Materials Science
More specific subject area:	Microfluidic double emulsion
Method name:	Two-step generation of monodisperse agarose-solidified double emulsions (w/w/o) excluding an inner oil barrier
Name and reference of original method:	Combination of high-throughput Microfluidics and FACS technologies to leverage the numbers game in natural product discovery http://doi.org/10.1111/1751–7915.13872
Resource availability:	Microfluidic hardware/software: https://www.dolomite-microfluidics.com/microfluidic-systems/%C2%B5encapsulator/

## Background

The field of microfluidics set new standards in miniaturization of biomedical and chemical research areas. Droplet-based microfluidics is used as a tool for small scale single-cell or cell culture analysis, chemical synthesis as well as high-throughput screening [1]. Achieved by dispersions of stabilized liquids within continuous immiscible fluids, thousands of emulsions are generated within minutes, which subsequently are used as micro compartments suitable for such experiments [2]. Beside simple water-in-oil (w/o) or oil-in-water (o/w) emulsions, more complex ones, carrying single or multiple emulsions that represent more compartments, enable the build-up of systems with higher complexity. However, the latter are more difficult to generate. Widely used are water-in-oil-in-water (w/o/w) and oil-in-water-in-oil (o/w/o) emulsions, which can be employed for applications such as drug delivery vehicles, cell carriers, barcoding of droplets, microscale sensors, and more [3]. Other combinations of phases such as w/w and w/w/w are challenging, however applicable using aqueous phases of different properties, e.g., density, viscosity, and refractive index [4,5]. With millions of emulsions generated within minutes, powerful high-throughput analysis and sorting tools are required to retrieve events of interest. Optofluidic setups [6,7], fluorescence-activated droplet sorter (FADS) [8,9] or commercially available multichannel fluorescence-activated cell sorter (FACS) [10,11] are such tools. They differ in the analyzable emulsion-types, sample throughput and sorting capabilities, assay readout, and biocompatibility.

**Fig. 1:**
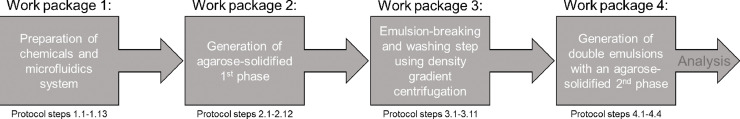
Overview of the complete workflow to generate agarose-solidified (w/w/o) double emulsions.

In this protocol (Fig. 1), we demonstrate the generation of agarose-solidified (w/w/o) double emulsions (diameter: ∼55 µm, volume: ∼87 pL) building on agarose-solidified (w/o) emulsions (first phase; diameter: ∼40 µm, volume: ∼33.5 pL) that are produced beforehand with the same microfluidic setup. The workflow includes an emulsion-breaking step of the first phase that results in the release of all compartments of the first phase into an aqueous phase. This is followed by a density-gradient washing step thereof to wash the first phase droplets and while working with e.g., cell cultures or bacteria to separate them from motile cells that did not get stuck within the agarose droplets. Subsequently, a second phase of liquid agarose is added. This procedure enables that both agarose phases are not compartmentalized by the fluorocarbon oil (Novec^TM^ 7500), which is stabilized with a surfactant (Pico-Surf^TM^ 1), necessary for emulsion stability. The emulsion-breaking step is essential, since the oil and surfactant are known to function as a barrier, which however is not completely understood until today, for several molecules with slow diffusion properties [12,13]. Moreover, with no diffusion barrier and the second phase to be added any time after incubation of the first phase, this method allows various applications for e.g., two-layer high-throughput cell culture and screening assays that rely on an intermediate washing step. This is supported by the use of a low-melting agarose that allows operations at low temperatures feasible for cell culture experiments. Applicable for e.g., fluorescence-activated cell sorting (FACS)-based technologies, a variable high-throughput readout of an envisaged assay, based on such double emulsions, is given. To the best of our knowledge, this is the first report of successful generated agarose-solidified double emulsions not separated by an inner oil barrier. Designated to run on the commercially available microfluidic ‘μEncapsulator System’ (Dolomite Microfluidics, Royston, UK), trained microfluidic users can easily implement this method in any lab.

## Overview of the method

Fig. 1 depicts the general procedure to generate agarose-solidified (w/w/o) double emulsion using the commercially available microfluidic ‘μEncapsulator System’ (Dolomite Microfluidics), including solution and microfluidics system preparation (work package 1), generation of agarose-solidified (w/o) emulsion (first phase, work package 2), an emulsion-breaking and washing step of the first phase (work package 3), and the generation of the final agarose-solidified (w/w/o) double emulsions (work package 4). Work package 3 is critical for this application, especially while working with cell cultures. The emulsion of the solidified agarose and the fluorocarbon oil Novec^TM^ 7500 stabilized with Pico-Surf^TM^ 1 (first phase) needs to be de-emulsified, since otherwise in the end both agarose phases would be separated by a diffusion barrier not fully understood [12,13]. The emulsion-breaking results in release of the first phase content into the aqueous phase that did not get stuck within the agarose, e.g., still motile cells. As a solution to separate agarose-solidified droplets of the first phase from other compartments, we designed a washing step based on density gradient centrifugation using Nycodenz®.

## Materials

### Chemicals

• Pico-Surf™ 1 (5% (w/w) in Novec™ 7500) (Sphere Fluidics, Cambridge, UK, prod. no. C022)
• Novec™ 7500 (Iolitec Ionic Liquids Technologies GmbH, Heilbronn, GER, prod. no. FL-0004-HP)
• Pico-Break™ 1 – Emulsion Breaking Solution (Sphere Fluidics, Cambridge, UK, prod. no. C081)
• SeaPrep agarose (Lonza, Basel, Switzerland, prod. no. 50,302)
• Nycodenz® (Axis-Shield Poc AS, Oslo, Norway, prod. no. 1,002,424)
• 2-Propanol (Honeywell International Inc., Morristown, New Jersey, US, prod. no. 34,965)

### Equipment

• Temperature control unit, Meros TCU-100 (TCU, Dolomite Microfluidics, Royston, UK, prod. no. 3,200,428)
• µEncapsulator Top Interface (Dolomite Microfluidics, Royston, UK, prod.no. 3,200,569)
• Linear Connector (Dolomite Microfluidics, Royston, UK, prod. no. 3,000,024)
• μEncapsulator 1 Sample Reservoir Chip (Dolomite Microfluidics, Royston, UK, prod. no. 3,200,444)
• μEncapsulator 1 - 2 Reagent Droplet Chip (50 μm etch depth), fluorophilic, (Dolomite Microfluidics, Royston, UK, prod. no. 3,200,445)
• Pressure pumps, Mitos P-Pump (Dolomite Microfluidics, Royston, UK, prod. no. 3,200,176)

○ Mitos Flow Rate Sensor (Dolomite Microfluidics, Royston, UK, prod. no. 3,200,098, 3,200,099)
○ Mitos Sensor Interfaces (Dolomite Microfluidics, Royston, UK, prod. no. 3,200,200)

• Four-way connector (Dolomite Microfluidics, Royston, UK, prod. no. 3,200,454)
• Air compressor Fengda AS-189
• High-speed CMOS camera PL-D721CU (Navitar Inc., Rochester, NY, USA)
• Stereomicroscope Stemi SV 11 (Carl Zeiss, Oberkochen, Germany)
• Halogen light source KL 2500 LCD (Schott AG, Mainz, Germany)
• Fluorescence microscope DM2000 LED equipped with a DFC450 C camera (Leica Microsystems, Wetzlar, Germany)
• Centrifuge 5810 R (Eppendorf AG, Hamburg, Germany)
• Centrifuge 5424 R (Eppendorf AG, Hamburg, Germany)
• Fridge
• Neubauer chambers (0.1 mm depth, Paul Marienfeld GmbH & CO. KG, Lauda-Königshofen, Germany)
• Adjustable 10, 100 and 1000 μL pipettes

### Consumables

• 1 mL NORM-JECT syringes (Henke-Sass, Wolf GmbH, Tuttlingen, Germany prod. no. 4010.200V0)
• 30 mL syringes (Becton, Dickinson and Company, Franklin Lakes, New Jersey, US, prod. no. 309,650)
• Needles – Sterican® Gr. 14, G 23 × 1 1/4″" / ø 0,60 × 30 mm, blue (B. Braun SE, Melsungen, Germany, prod. no. 4,657,640)
• 15 mL centrifuge tubes (Greiner Bio-One International GmbH, Frickenhausen, Germany, prod. no. 188,261)
• 0.2 µm CA syringe filter (Corning Inc., Corning, NY, USA, prod. no. 431,224)
• 1.5 mL reaction tubes (SARSTEDT AG & Co. KG, Nümbrecht, Germany)
• Sterile pipette tips
• Microorganisms used as showcases in this study:

○ Environmental microorganisms isolated in our previous study [13]
○ *E. coli* DH5α pFU95 (expressing *gfp_mut3.1_*)
○ *E. coli* DH5α pFU96 (expressing *dsRed2*)

### Software

• Dolomite Flow Control Center (Dolomite UK, prod. no. 3,200,475)
• Pixelink Capture Software (Navitar Inc., Rochester, NY, USA)
• Leica Application Suite v4.8.0 (Leica Microsystems CMS GmbH, Heerbrugg, Switzerland)

## Experimental protocol

In this section each work package and therein the individual tasks (protocol steps) are described step-wise and in detail.

1. *Work package – Preparation of chemicals and microfluidic system*

**Fig. 2:**
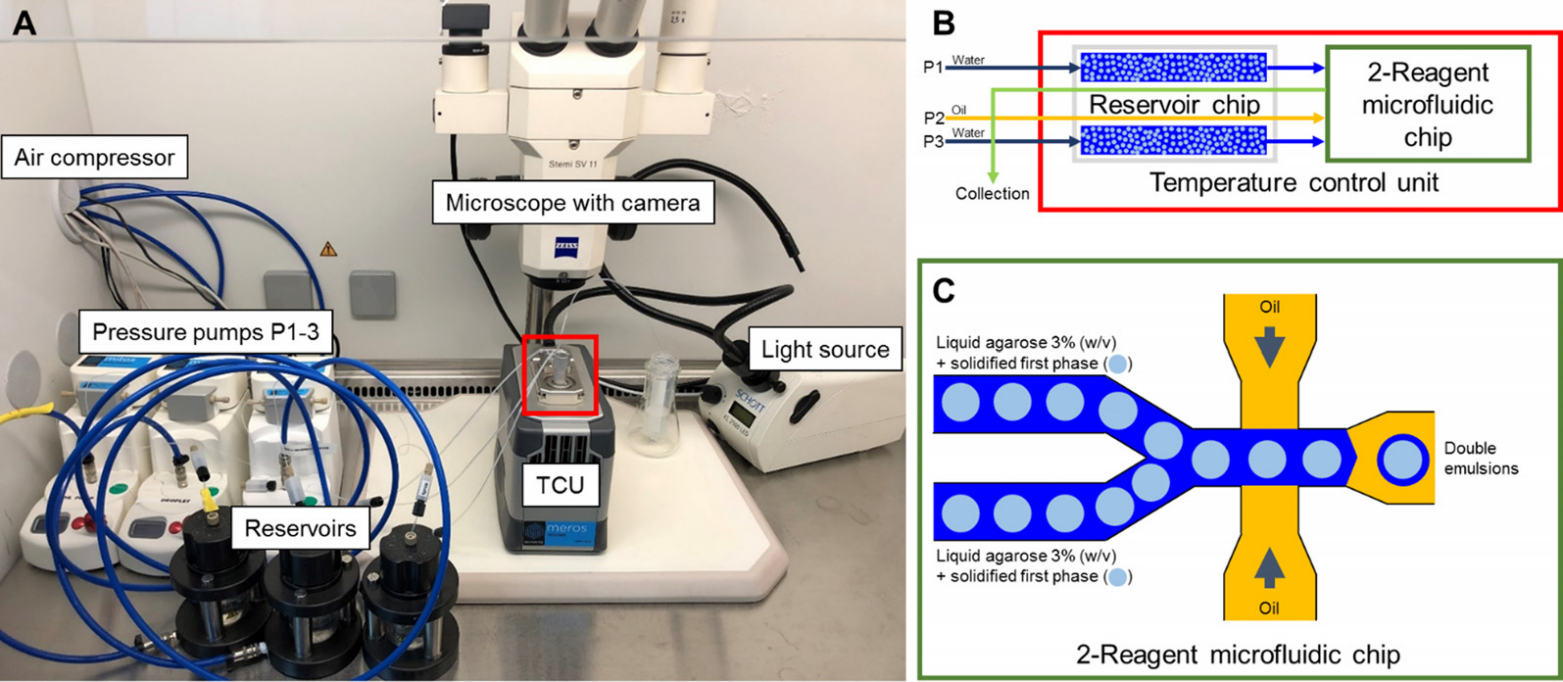
Overview of the hardware setup (a), the temperature control unit (b) and the 2-Reagent microfluidic chip (c) used for the generation of the agarose-solidified 1st and 2nd phase.

The commercially available microfluidic ‘µEncapsulator System’ (Dolomite Microfluidics) is very user-friendly (Fig. 2). The assembly of all parts, the cleaning and sterilization of chips as well as the troubleshooting of blockages is described in the ‘μEncapsulator System User Manual’ (https://www.dolomite-microfluidics.com/support/downloads/). Prior to each experiment, prepare and perform the following chemicals and steps:

1.1 Prepare 20 mL Novec™ 7500 containing 1% (w/w) Pico-Surf™ 1 (mix 4 mL Pico-Surf™ 1 5% (w/w) in Novec™ 7500 with 16 mL Novec™ 7500).
1.2 Prepare 40 mL water.
1.3 Prepare 3% (w/v) SeaPrep agarose in water (3 g SeaPrep in 100 mL water) and autoclave. **NOTE** Add a magnetic stirrer because after autoclaving the agarose is not dissolved well. Stir it at 60 °C until a homogeneous solution is obtained.
1.4 Prepare 20% and 30% (w/v) Nycodenz® solution in water (10 g and 15 g Nycodenz® in 50 mL water each).
1.5 Prepare 70% isopropanol (HPLC grade).

**Important** Filter all solvents through 0.2 µm CA syringe filters to prevent blockage of microfluidic channels.
Microfluidic system (Fig. 2a):

1.6 Fill reservoirs with water (pump 1 and 3) and Novec™ 7500 with 1% (w/w) Pico-Surf™ 1 (pump 2).
1.7 Turn on the air compressor for pressure supply and operate it at 500–700 kPa.
1.8 Turn on all three pressure pumps, the TCU (temperature control unit), light source, and computer.
1.9 Open the Flow Control Center (FCC) Software (Dolomite Microfluidics) and Pixelink Capture Software (Navitar).

**IMPORTANT** The FCC software needs to be opened after the hardware is switched on to ensure connection.

1.10 Set the temperature of the TCU to 30 °C to prevent solidification of the low-melting agarose during encapsulation.
1.11 Priming the system (Fig. 2b) is done with the Linear Connector connected to the reservoir chip. Apply flow rates of 5 µL/min on each channel for 5 min. This is necessary to prime all channels with their respective fluids and to push e.g., air bubbles out of the system.
1.12 Set the flow rates to 0 µL/min and disconnect the Linear Connector. **NOTE** This ensures no backflow of fluids into their reservoirs without using valves.
1.13 Empty both sample reservoirs on the sample reservoir chip.

→ The system is now ready to be used for the generation of the first phase or for the generation of double-emulsions in the second step.

2. *Work package – Generation of agarose-solidified 1st phase*

We previously used the following protocol steps for encapsulation and cultivation of environmental microorganisms [13]. To obtain the here described agarose-solidified (w/w/o) double emulsions, it was necessary to use SeaPrep agarose instead of the previously used SeaPlaque one. Agarose-solidified (w/w/o) double emulsions were not possible to be obtained from a first phase produced with SeaPlaque agarose, probably due to different material properties.

2.1. Prepare the loading suspension to obtain a first phase of 1.5% (w/v) agarose droplets in a 1.5 mL reaction tube as following:
• 200 µL 3% (w/v) SeaPrep in water
• 200 µL water

**NOTE** Working with cell cultures, the water can be exchanged with the cells in their specific medium and other components. It is also possible to prepare the agarose with medium in advance to prevent dilution after mixing the liquid agarose and the water phase (cells + medium).

2.2 Vortex the solution for 10 s.
2.3 Load ∼90 µL into each reservoir of the reservoir chip.
2.4 Pipet 5 µL of Novec^TM^ 7500 post each loaded sample. **NOTE** This keeps the loaded sample separated from the water pushing it towards the 2-Reagent Droplet Chip (Fig. 2c).
2.5 Close the lid and connect the Linear Connector.
2.6 Set the flow rates for both pumps 1 and 3 supplying the loaded sample towards the 2-Reagent microfluidic chip to 2 µL/min and the flow rate for the oil (pump 2) to 40 µL/min. **NOTE** The pressure of the oil pump will be in the range of 350–550 mbar and of the water pumps between 550 and 800 mbar. A single pump should not exceed 1200 mbar, otherwise the channel of this pump is blocked. For the troubleshooting of blockages see the ‘μEncapsulator System User Manual’ (https://www.dolomite-microfluidics.com/support/downloads/).
2.7 Wait 2–3 min for pressures to stabilize and then start to collect the generated droplets in a 1.5 mL reaction tube for about 25 min. **NOTE** A pressure drop indicates an emptied reservoir and therefore the end of the encapsulation.
2.8 Set the flow rate of all three pumps to 0 µL/min.
2.9 Incubate the first phase at 8 °C for 20 min to solidify the liquid agarose-in-oil emulsions.
2.10 Disconnect the Linear Connector and empty both reservoirs of the reservoir chip.
2.11 Clean and sterilize the reservoir chip and the 2-Reagent microfluidic chip as following:
• To solve remaining residues pipet water up and down in each reservoir a few times followed by 70% isopropanol.
• Load both reservoirs with 70% isopropanol.
• Close the lid and connect the Linear Connector.
• Set all pumps to 7 µL/min and run the system for 10 min.
• Load both reservoirs with water.
• Close the lid and connect the Linear Connector.
• Set all pumps to 7 µL/min and run the system for 10 min.
  2.12 After cleaning and sterilization, stop the flow of all three pumps. **NOTE** For long term storage all channels are dried/emptied.

→ At this point the microfluidic system is on hold. A shut down can be done by disconnecting the power of all individual parts, releasing the pressure from the air compressor and shutting down the computer.

3. *Work package – Emulsion-breaking and washing step using density gradient centrifugation*

**Fig. 3:**
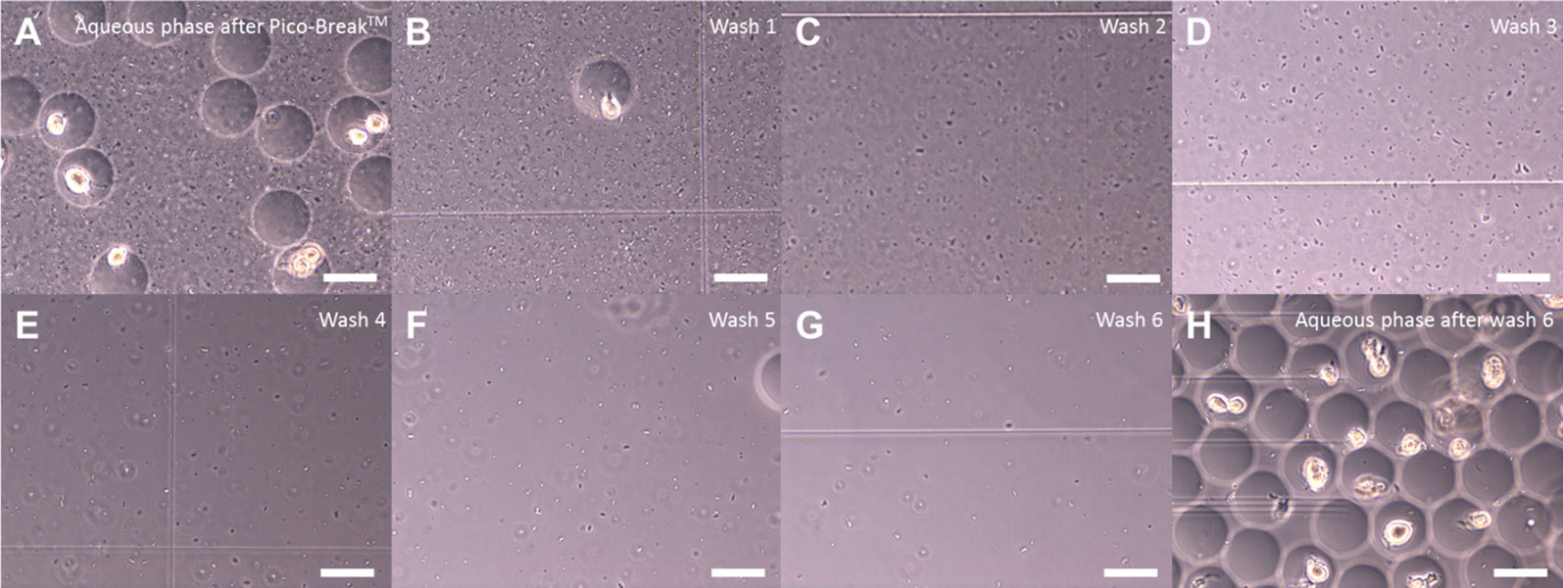
Aqueous phase before (a), in-between (b-g) and after six washing steps consisting of centrifugation and supernatant exchange. Scale bar: 40 µm.

**Fig. 4:**
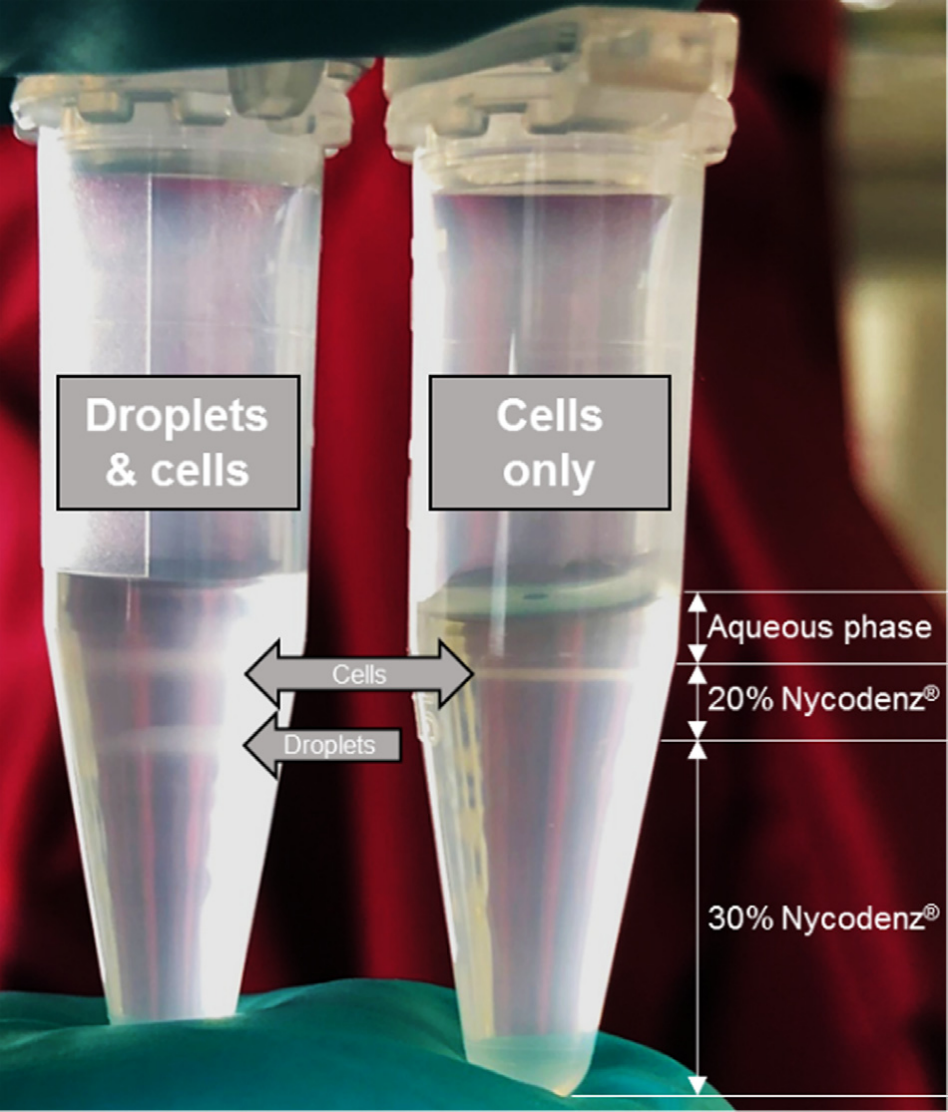
Successful separation of free-swimming cells and agarose-solidified droplets of the first phase using density gradient centrifugation based on Nycodenz®.

**Fig. 5:**
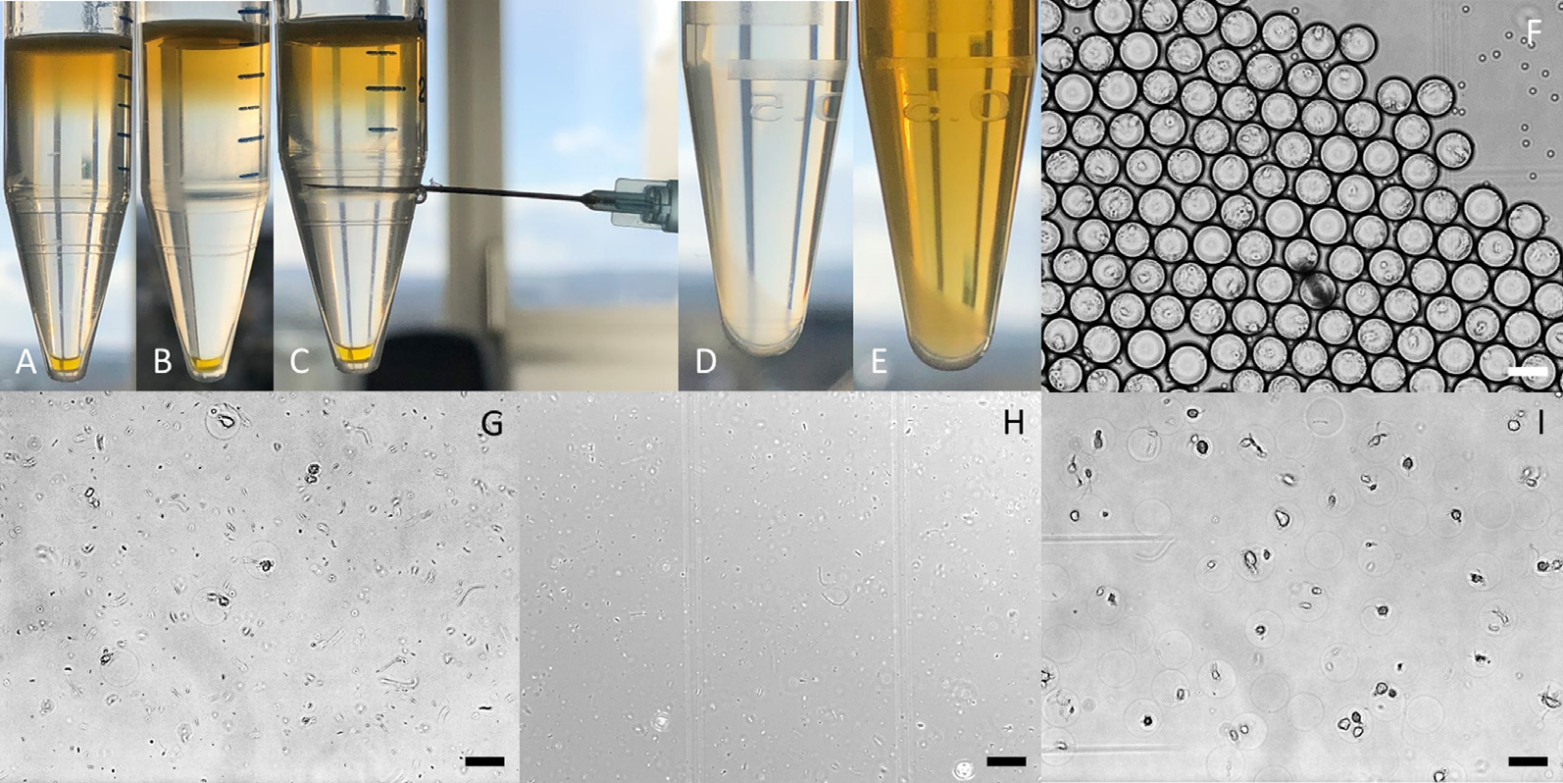
Droplet-layer extraction after density gradient centrifugation (before (a) and after (b) centrifugation, pierced needle on droplet-layer level (c), milky droplet pellet in water (d), milky droplet pellet in medium (e)). Microscopic picture of the w/o emulsion of the first phase before (f) and after Pico-Break^TM^ 1 treatment (g), the cell-layer (h) and the droplet-layer (i) after density gradient centrifugation. Scale bar: 40 µm.

The generation of agarose-solidified double emulsions with both agarose phases not separated by the surfactant (Pico-Surf^TM^ 1) stabilized fluorocarbon oil (Novec^TM^ 7500) requires an emulsion-breaking step of the first phase. Otherwise, both agarose phases are oil-separated – a barrier probably unwanted for some high-throughput assays, since diffusion properties for several molecules are not completely understood today [12,13]. The emulsion-breaking step is performed using Pico-Break^TM^ 1, according to the manufacturer's instruction with minor adaptations (https://spherefluidics.com/wp-content/uploads/2019/03/Pico-Break_User-Guide-March-2019.pdf). This results in non-oil/surfactant-separated agarose-solidified droplets in an aqueous phase. Separated environments are still preserved, e.g., for cell cultures, or as in our case bacteria, that have been encapsulated and got stuck in the solidified agarose of the first phase. However, while working with bacteria, we observed a huge problem of free swimming non-agarose-attached cells in the aqueous phase after emulsion-breaking (Fig. 3a). Applying this mixed aqueous phase to the second encapsulation to add the outer agarose layer, resulted in cross contamination of all solidified droplet-compartments by free swimming cells. A simple washing step by centrifugation and supernatant exchange resulted in a reduction of free swimming cells (Fig. 3b–g); however, even after six repetitions a few of them were observed in the aqueous phase (Fig. 3h). Therefore, a density gradient centrifugation protocol based on Nycodenz® (Figs. 4, 5a–i) was established to completely separate the free swimming cells (Fig. 5h) from the agarose-solidified droplets (Fig. 5i) containing non-motile cells with the following protocol steps:

3.1 Take the 1.5 mL reaction tube containing the first phase (agarose-solidified droplets and oil, Fig. 5f) and remove as much of the Pico-Surf™ 1 oil (bottom layer) as possible using a standard pipet. **NOTE** This reduces the amount of Pico-Break^TM^ 1 necessary to break the w/o-emulsion.
3.2 The droplet layer has a volume of approximately 100 µL. Add 250 µL Pico-Break^TM^ 1 and gently agitate the mixture by inverting. **NOTE** The solution turns orange and starts to disperse.
3.3 Centrifuge the sample at 1000 × *g* for 1 min to disperse the aqueous (clear) and fluorous (orange) phase.
3.4 Tilt 1.5 mL reaction tube to an angle of 45° and remove most of the orange fluorous phase using a standard pipet.

Add 500 µL water to the remaining solution in the 1.5 mL reaction tube and transfer it to a 15 mL centrifuge tube. **NOTE** While working with cell cultures, the water can be exchanged with specific medium and other components.

3.5 Carefully pipet 2 mL 20% Nycodenz® solution underneath this aqueous phase.
3.6 Carefully pipet 1 mL 30% Nycodenz® solution underneath the 20% Nycodenz® solution. Fig. 5a depicts how the solution should look like at this step.
3.7 Centrifuge at 2000 × *g* for 1 min. **IMPORTANT** Do not centrifuge at higher speed or elongate this step, otherwise the cell-layer will not be separated from the droplet-layer (Fig. 5b).
3.8 To extract the droplet-layer, place a needle on top of a 1 mL syringe, pierce slowly through the centrifuge tube beneath the droplet-layer, which is situated on top of the 30% Nycodenz® solution, and pull out the droplets (Fig. 5c).
3.9 Transfer the clear content to a fresh 1.5 mL reaction tube.
3.10 Centrifuge the solution at 1000 × *g* for 1 min and discard the supernatant until 200 mL remain. **NOTE** The droplet-layer is hard to spot in a clear solution (Fig. 5d).
3.11 Wash the droplet-layer by adding 1000 mL water (or medium), centrifugation at 1000 × *g* for 1 min (Fig. 5e) and discard the supernatant until 100 mL are left.

→ At this point, the emulsion of the first phase is broken, the droplet-layer is washed and potentially free-swimming cells are discarded. The droplet-layer can now directly be used to be encapsulated a second time to add the second agarose layer.

4. *Work package – Generation of double emulsions with an agarose-solidified 2nd phase*

This work package is highly similar to the second one, since the same system and chips are used. The main difference for the production of double emulsions is that a different flow rate on the oil pump (pump 2) is applied.

4.1 Prepare the loading suspension to obtain double emulsions in a 1.5 mL reaction tube as following:

• 100 µL 3% (w/v) SeaPrep in water
• ∼100 µL droplet-layer of the first phase after work package 3 (oil-free and washed)

4.2 Perform the previously described protocol Steps 2.2 to 2.5.
4.3 Set the flow rates for both pumps 1 and 3 supplying the loaded sample towards the 2-Reagent microfluidic chip to 2 µL/min and the flow rate for the oil (pump 2) to 15 µL/min. **NOTE** The pressure of the oil pump will be in the range of 450–650 mbar and of the water pumps between 800 and 1100 mbar. A single pump should not exceed 1400 mbar, otherwise the channel of this pump is blocked. For the troubleshooting of blockages see the ‘μEncapsulator System User Manual’ (https://www.dolomite-microfluidics.com/support/downloads/).
4.4 Perform the previously described protocol Steps 2.7 to 2.12.

→ At this point, agarose-solidified double emulsions (w/w/o) without an inner oil barrier are generated and can be analyzed, e.g., using FACS, as previously described for agarose-solidified droplets of the first phase [13].

## Method validation

**Fig. 6:**
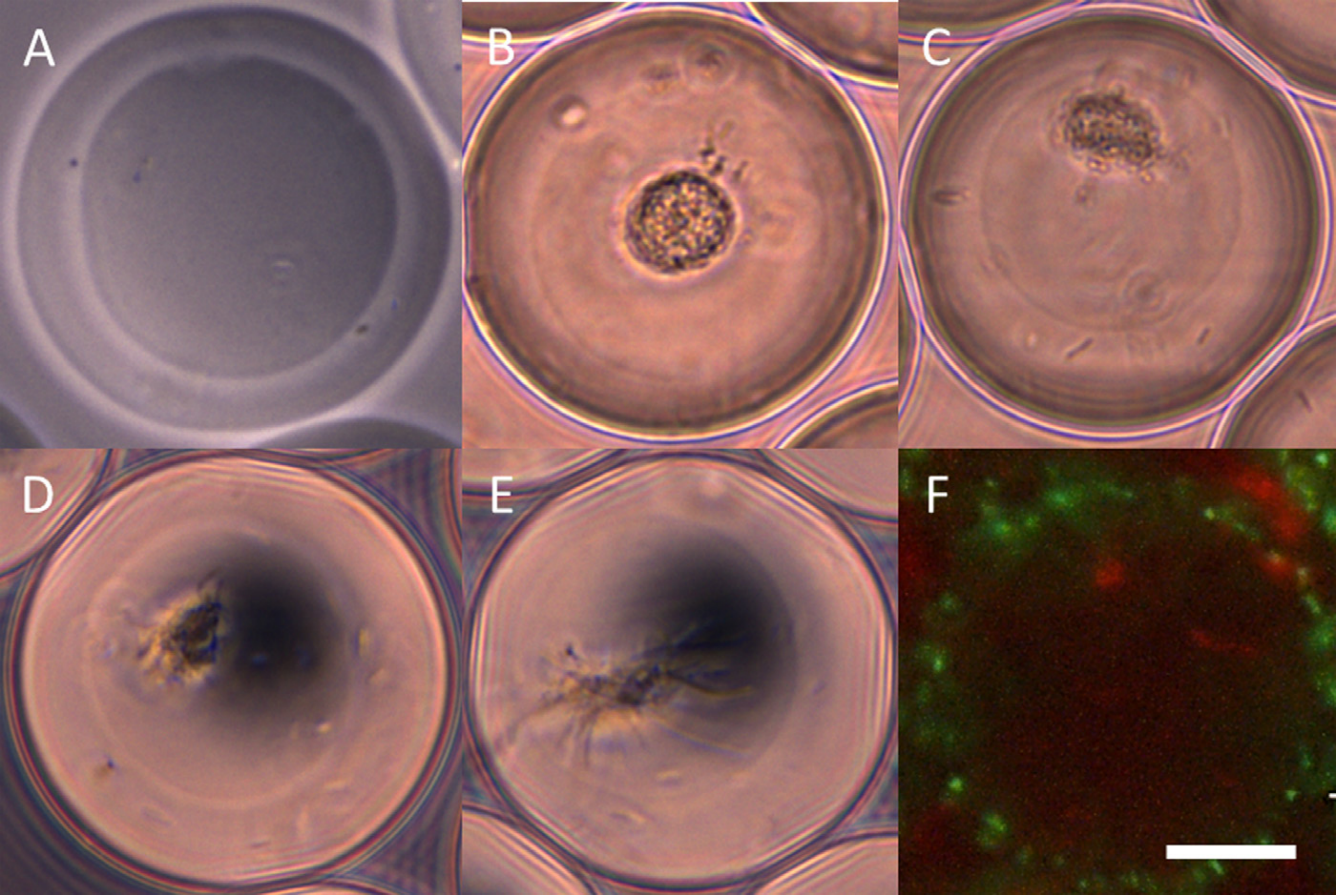
Examples of agarose-solidified double emulsions with both phases empty (a), first phase carrying a microcolony of *E. coli* DH5α derived from a single cell (b–e), and one with dsRed producing *E. coli* DH5α pFU96 cells in the first phase and GFP producing *E. coli* DH5α pFU95 cells in the outer layer (f). Scale Bar: 20 µm.

Some agarose-solidified double emulsion examples are shown in Fig. 6. They are ∼55 µm in diameter with a volume of ∼87 pL and they can be empty (Fig. 6a) or e.g., carry bacteria. In our case we did experiments with first phases carrying single *E. coli* DH5α cells, incubated for a few hours and engulfed with a second agarose layer (Fig. 6b–e). An encapsulation of dsRed producing *E. coli* DH5α pFU96 cells in the first phase and GFP producing *E. coli* DH5α pFU95 cells in the outer layer (Fig. 6f) shows how cells are distributed in both compartments. Usually the microfluidics setup and all chemicals are prepared within half an hour. A single encapsulation generating the first or second phase takes with preparation about 45 min. To safe time between experiments, the emulsion-breaking and washing step on the next sample can be applied while the microfluidics system runs with another sample. Therefore, several encapsulation are possible on one day.
